# Carotid Intima-Media Thickness in Surgically or Conservatively Managed Patients With Primary Hyperparathyroidism

**DOI:** 10.1210/clinem/dgae053

**Published:** 2024-02-12

**Authors:** Vincenzo Carnevale, Flavia Pugliese, Cristina Eller-Vainicher, Antonio S Salcuni, Luciano Nieddu, Iacopo Chiodini, Alfredo Scillitani

**Affiliations:** Unit of Internal Medicine, “Casa Sollievo della Sofferenza” Hospital, IRCCS, 71013 San Giovanni Rotondo, FG, Italy; Unit of Endocrinology, “Casa Sollievo della Sofferenza” Hospital, IRCCS, 71013 San Giovanni Rotondo, FG, Italy; Endocrinology Unit, Fondazione IRCCS “Ca’ Granda Ospedale Maggiore Policlinico, 20122 Milan, Italy; Unit of Endocrinology and Metabolism, University-Hospital S. Maria Della Misericordia, 33100 Udine, Italy; Department of Humanistic and International Social Sciences, UNINT University, 00147 Rome, Italy; Unit of Endocrinology, Ospedale Niguarda Cà Granda, 20112 Milan, Italy; Unit of Endocrinology, “Casa Sollievo della Sofferenza” Hospital, IRCCS, 71013 San Giovanni Rotondo, FG, Italy

**Keywords:** primary hyperparathyroidism, intima-media thickness, plaque, parathyroidectomy

## Abstract

**Context:**

Current evidence of cardiovascular (CV) risk in primary hyperparathyroidism (PHPT) is still inconsistent.

**Objective:**

To prospectively investigate changes of early atherosclerosis in patients with PHPT undergoing parathyroidectomy (PTx) or conservative management, according to consensus criteria.

**Methods:**

Biochemical parameters of PHPT, CV risk factors (systolic and diastolic blood pressure, cholesterol [total, high-density, and low-density], triglyceride, HbA1c, HOMA-IR), and carotid intima-media thickness (IMT) and plaque were assessed in 52 consecutive postmenopausal PHPT patients both at baseline and ≥ 24 months after surgery (PTx, n = 22) or conservative management (non-PTx, n = 30).

**Results:**

At baseline, PTx and non-PTx showed comparable age, BMI, renal function, and 25(OH)D levels, and did not differ for CV risk factors, IMT and plaques, or for prevalence of smoking, diabetes mellitus, or antihypertensive or statin therapy, while all parameters characterizing PHPT differed. Follow-up duration in PTx was longer than in non-PTx (*P* = .004). Parameters characterizing PHPT significantly improved ≥ 24 months after surgery, whereas in non-PTx serum phosphate slightly decreased and parathyroid hormone increased. Systolic and diastolic blood pressure increased at follow-up in both groups, while other CV risk factors did not significantly vary. In PTx, IMT did not significantly vary after surgery (0.85 ± 0.14 to 0.89 ± 0.22 mm, *P* = .366), whereas it significantly increased in non-PTx (0.80 ± 0.18 to 0.93 ± 0.23 mm, *P* = .008), even adjusting for blood pressure. Plaque prevalence and incidence did not significantly differ in the 2 groups.

**Conclusion:**

Our results suggest that in postmenopausal patients with PHPT, subclinical atherosclerosis could be halted by PTx, whereas it worsens over time in nonoperated patients with milder disease.

Primary hyperparathyroidism (PHPT), classically regarded as a skeletal and renal disease, now quite commonly presents as a mild or asymptomatic condition. Recent association studies, mainly focused on mild or asymptomatic PHPT, investigated possible nonclassical characteristics of the disease, such as neuromuscular, neuropsychiatric and cognitive, and cardiovascular (CV) features (1). Among these, clinical and subclinical CV manifestations deserve utmost interest, due to the impact of CV diseases on general health. According to these studies, PHPT seems to be associated with increased CV morbidity and mortality relative to the general population. This could relate to its possible associations with many CV risk factors, such as arterial hypertension, dyslipidemia, impaired glucose homeostasis, altered ventricular mass and arrhythmic risk, and altered function and structure of coronary and peripheral arteries (1-3). However, available relevant data to date are not uniform and the evidence of improved CV events and CV risk factors after parathyroidectomy (PTx) is still inconsistent and does not allow inference of causality (1, 4).

Ultrasonographic assessment of the carotid arteries enables the measurement of intima-media thickness (IMT) and to identify plaques. It has been demonstrated that the ultrasound-measured distance between the intimal-luminal and the media-adventitial interfaces of the carotid far wall overlaps with the measurement obtained on pathologic examination (5). Accordingly, carotid IMT and plaques have been widely utilized as an easy-to-obtain, sensitive, and reproducible hallmark of early-stage atherosclerosis (6, 7). Being both age- and comorbidity-dependent, IMT measurement has been utilized in large studies and meta-analyses which showed that carotid IMT and plaque, possibly together with other risk factors, may predict future CV events (6, 8-11). Thus, the assessment of carotid IMT and plaques may be a useful tool to detect and monitor early-stage and preclinical atherosclerosis, and at this purpose it has been utilized to investigate CV risk in patients with PHPT (12-25). Despite this wealth of data, results are often conflicting and evidence on this matter remains obscure. This is likely due to the relevant differences among studies in terms of sample size, gender composition, age, cross-sectional or longitudinal design, and in the latter case, the follow-up duration. Therefore, in order to avoid both the possible bias related to the known gender difference in the risk factors and pattern of CV disease (26), and those attaining PHPT epidemiology (27), we chose to investigate subclinical atherosclerosis only in postmenopausal female patients with PHPT. With this purpose, we prospectively investigated carotid intima-media thickness and plaques, together with other main CV risk factors, in a sample of 52 consecutive postmenopausal women with PHPT.

## Methods

### Patients

**Figure 1.**
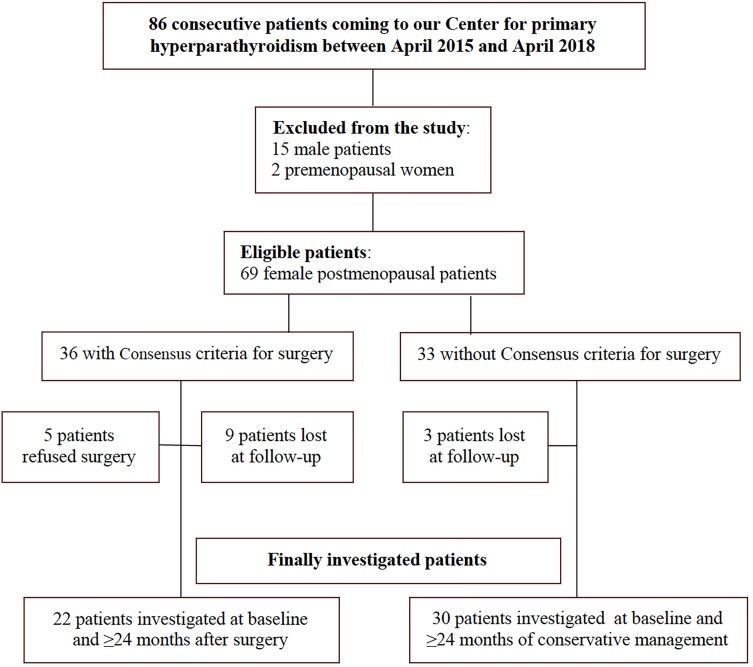
Flow-chart of the study.

In this prospective evaluation we chose to include exclusively postmenopausal women. Therefore, out of 86 consecutive patients with PHPT coming to our Endocrinology Unit between April 2015 and April 2018, we excluded 15 male patients and 2 premenopausal women. In all patients, history, physical exam, assessment of calcium homeostatic parameters, lipid profile, insulin resistance, and glycated hemoglobin, as well as assessment of the carotid arteries by M-mode ultrasonography was carried out at baseline. According to the current consensus criteria and those released in 2022 (28), 36 patients were referred for surgery and 33 were conservatively managed. A clinical, laboratory, and ultrasonographic re-evaluation was performed after ≥ 24 months in all patients, except in 5 women who met surgical criteria but refused intervention, as well as in 9 patients who had undergone PTx and 3 conservatively managed patients who were lost at follow-up. Therefore, the finally analyzed sample of 52 postmenopausal women with PHPT included 22 patients assessed before and after surgery (PTx group), and 30 conservatively managed (non-PTx group) also evaluated twice (with the same planned timing) (Fig. 1). All subjects gave informed consent and the study was approved by the local ethics committee.

All the included patients were investigated twice with the same laboratory protocol. A morning fasting blood sample and a concomitant 24-hour urine collection were obtained. Serum and urinary creatinine, calcium, and phosphate, as well as serum alkaline phosphatase total activity, albumin, total and high-density lipoprotein (HDL)-cholesterol, triglyceride, and glucose levels were measured by a multichannel autoanalyzer (Hitachi 747, Japan). Low-density lipoprotein (LDL)-cholesterol was calculated by the Friedwald formula (29). Glycosylated hemoglobin (HbA1c) was measured by high-performance liquid chromatography (BIO-RAD Laboratories, Segrate, MI, Italy). Glomerular filtration rate (eGFR) was estimated by the Chronic Kidney Disease–Epidemiology Collaboration (CKD-EPI) equation (30). Serum ionized calcium (corrected to pH 7.4) was assessed by ion-specific electrode. Serum parathyroid hormone (PTH) and 25-hydroxyvitamin D [25(OH)D] levels were assessed by immunochemiluminometric assays (Liason 1-84 PTH, Diasorin Inc., Stillwater, MN, USA; RRID:AB_2811286, and Diasorin, Stillwater, MN, USA; RRID:AB_2811287), as well as serum insulin (ADVIA Centaur Insulin (IRI), Siemens Healthcare Diagnostics, NY, USA; RRID:AB_2909499) and serum β-CrossLaps (β-C-terminal telopeptide; β-CTx,) (Roche Diagnostics GmbH, Mannheim, Germany; RRID:AB_2905599).

The IMT and plaque were assessed twice (separated by at least 24 months). All patients were examined by a single trained operator, who was blinded to their clinical and biochemical characteristics. High-resolution B-mode ultrasonography was performed with the patients in the supine position using a 7.5-MHz linear probe (MyLab 30, Esaote, Genova, Italy). The distance between the intimal-luminal and the media-adventitial interfaces of the far wall (IMT) of each carotid artery was measured. The reported values were obtained by averaging 3 separate measurements along a tract of 10 mm of the far wall of each common carotid artery, at least 5 mm from the carotid bulb, only in stenotic-free segments. The values from the left and right side were averaged. Longitudinal and cross-sectional views were used to visualize focal atherosclerotic lesions, and a focal thickening of >1.5 mm was defined as a carotid plaque (31). Plaque results are presented as absent/present (Y/N). Interscan and intra-observer reproducibility was good, with an intraclass correlation coefficient of 0.94. The intra-observer intra-session coefficient of variability was 2.6%.

### Statistical analysis

Data are presented as mean ± SD. After normality testing, basal continuous values of the PTx and non-PTx groups were compared by unpaired Student *t* test or the Mann–Whitney test, as appropriate, while nominal or ordinal values were compared by the Chi-square test or the Fisher exact test. Basal and follow-up continuous values of either PTx or non-PTx were compared by paired *t* test or Wilcoxon test for paired samples, as appropriate, while the prospective ordinal parameters were compared by the McNemar test. Test results of both PTx and non-PTx patients for IMT were also corrected for systolic and diastolic blood pressure values, by general linear modeling. A *P* value of lower than .05 was considered significant.

## Results

At the initial evaluation, PTx and non-PTx patients did not differ for age (63.24 ± 7.72 vs 61.10 ± 8.14 years, respectively; *P* = .350) and body mass index (27.96 ± 4.61 vs 27.26 ± 4.53 kg/m^2^, respectively; *P* = .592). Four patients of the PTx group (18.2%) and 1 of the non-PTx group (3.3%) were smokers. Further, 14 (63.0%) PTx and 12 (40.0%) non-PTx patients were treated for arterial hypertension; 3 (13.6%) PTx and 2 (6.6%) non-PTx patients had a diagnosis of type 2 diabetes mellitus; and 4 (18.2%) PTx and 6 (20.0%) non-PTx patients received statin therapy. Such prevalences did not differ between PTx and non-PTx patients. As shown in the Table 1, patients who were candidates for PTx also did not differ at baseline from those of the non-PTx group for serum creatinine, eGFR, and serum 25(OH)D levels. However, in line with the current consensus (28), baseline biochemical values of patients who met criteria for PTx highly significantly differed from those of patients undergoing conservative management for all the biochemical parameters characterizing PHPT. At the initial assessment, both CV risk factors (systolic and diastolic blood pressure, cholesterol [total, HDL, and LDL], triglyceride, HbA1c, and homeostasis model assessment of insulin resistance [HOMA-IR] values) and indexes of vascular involvement (IMT and plaques) did not differ between PTx and non-PTx patients.

**Table 1.** Main biochemical parameters of primary hyperparathyroidism at T0 (baseline evaluation) and T1 (after surgery or conservative follow-up) of the 22 PHPT patients who underwent surgery (PTx) and of the 30 nonoperated patients (non-PTx)

	PTx at T0	PTx at T1	*P*	Non-PTx at T0	Non-PTx at T1	*P*
sCr (mg/dL)	0.70 ± 0.28	0.72 ± 0.25	.373	0.66 ± 0.12	0.64 ± 0.10	.247
eGFR (mL/min)	88.67 ± 20.60	85.78 ± 22.33	.692	93.19 ± 9.57	95.50 ± 12.00	.416
iCa (mmol/L)	1.53 ± 0.14	1.19 ± 0.08	**<.001**	1.39 ± 0.05*	1.40 ± 0.07	.568
sP (mg/dL)	2.60 ± 0.42	3.68 ± 0.62	**<**.**001**	2.94 ± 0.54°	2.75 ± 0.51	.**025**
s25(OH)D (ng/dL)	16.63 ± 7.65	30.10 ± 14.94	**<**.**001**	20.50 ± 8.81	24.55 ± 9.41	.056
sPTH (pg/mL)	212.07 ± 90.96	46.78 ± 21.92	**<**.**001**	117.15 ± 50.42*	134.72 ± 52.26	.**008**
sALP (U/L)	109.05 ± 46.16	76.30 ± 28.76	**<**.**001**	91.93 ± 26.97	100.73 ± 29.94	**<**.**001**
sCTx (ng/mL)	1.02 ± 0.45	0.46 ± 0.27	**<**.**001**	0.72 ± 0.31°	0.78 ± 0.25	.083
uCa (mg/day)	365.49 ± 163.06	138.87 ± 93.70	**<**.**001**	264.45 ± 100.01°	272.40 ± 84.33	.480
TmPO_4_/GFR	2.27 ± 0.63	3.03 ± 0.44	**<**.**001**	2.85 ± 0.69°	2.73 ± 0.74	.516
SBP (mmHg)	125.38 ± 10.20	132.18 ± 16.02	.**034**	121.87 ± 11.96	131.67 ± 14.16	**<**.**001**
DBP (mmHg)	71.86 ± 8.46	76.80 ± 11.13	.**017**	74.40 ± 8.92	79.67 ± 7.65	**<**.**001**
T-Chol (mg/dL)	197.67 ± 34.07	188.35 ± 41.03	.609	206.83 ± 32.26	202.33 ± 38.88	.379
HDL-C (mg/dL)	61.40 ± 16.34	60.19 ± 14.46	.934	59.26 ± 16.23	62.30 ± 16.38	.114
LDL-C (mg/dL)	114.24 ± 32.85	108.26 ± 36.15	.707	128.56 ± 37.67	118.17 ± 36.37	.069
Tryg (mg/dL)	95.27 ± 36.98	94.00 ± 27.90	.871	105.37 ± 45.82	109.07 ± 48.89	.687
HbA1c (%)	5.70 ± 1.06	6.06 ± 0.90	.064	5.64 ± 0.59	5.79 ± 0.66	.083
HOMA-IR	2.23 ± 1.21	2.45 ± 2.08	.963	2.06 ± 1.24	3.02 ± 3.98	.218
IMT (mm) ^§^	0.85 ± 0.14	0.89 ± 0.22	.366	0.80 ± 0.18	0.93 ± 0.23	.**008**
Plaques ^§^ (Y/N)	9/22 (40.91%)	10/22 (45.45%)	1	8/30 (26.67%)	11/30 (36.67%)	.242

Data are expressed as mean ± SD. The significance for comparison of baseline results is expressed by symbols: *P < 0.001; °*P* ≤ 0.01 (vs PTx basal values), whereas the columns of *P* values refer to the comparisons of T0 and T1 results.

Abbreviations: DBP, diastolic blood pressure; eGFR, glomerular filtration rate estimated through the CKD-EPI equation; HbA1c, glycosylated hemoglobin; HDL-C, serum high-density lipoprotein cholesterol; HOMA-IR, homeostasis model assessment of insulin resistance; iCa, ionized calcium; IMT, intima-media thickness; LDL-C, serum low-density lipoprotein cholesterol; plaques, carotid atherosclerotic plaques; s25(OH)D, serum 25-hydroxy vitamin D; sALP, serum alkaline phosphatase total activity; SBP, systolic blood pressure; sCr, serum creatinine; sCTx, serum crosslaps; sP, serum phosphate; sPTH, serum parathyroid hormone; T-Chol, total serum cholesterol; TmPO_4_/GFR, tubular mass of phosphate/GFR ratio; Tryg, serum tryglicerides; uCa, 24 hours urinary calcium.

**Figure 2.**
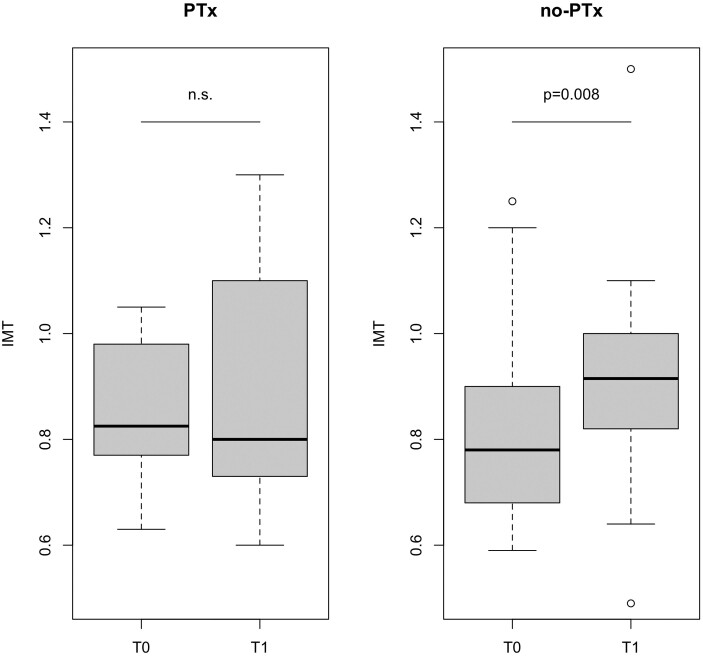
Box plot illustrating the intima-media thickness values at baseline assessment (T0) and at ≥ 24 months (T1) after surgery or conservative management, in operated (left panel) or nonoperated (right panel) patients with primary hyperparathyroidism.

All PTx patients underwent successful surgery, followed by normalization of serum calcium and PTH levels. Follow-up duration differed between groups; the mean time elapsed after surgery (32.59 ± 6.59 months) was significantly longer (*P* = .004) than the follow-up duration of non-PTx patients (28.17 ± 3.23 months). As expected (Table 1), in patients who underwent PTx, all parameters reflecting parathyroid hyperfunction and increased bone turnover were highly significantly improved at the follow-up check carried out ≥ 24 months after surgery, whereas their blood pressure values were slightly increased. Instead, in conservatively managed PHPT patients there was a slight but significant decrease of serum phosphate and increase of PTH levels. Both systolic and diastolic blood pressure values were also significantly increased at the follow-up visit in non-PTx PHPT patients. The other CV risk factors (total-, HDL-, and LDL-cholesterol; triglyceride; HbA1c; and HOMA-IR values) did not significantly change in both groups of patients, which in turn diverged for IMT changes. IMT values did not significantly vary after surgery, whereas it turned out to be highly significantly increased at follow-up in nonoperated PHPT patients (Fig. 2). After adjustment for both systolic and diastolic blood pressure values, IMT changes were still not significant in PTx, but they remained significant in non-PTx patients (*P* = .022 for both systolic and diastolic blood pressure). Plaque prevalence and incidence did not significantly differ in the 2 groups.

## Discussion

Our current results suggest that in postmenopausal female patients with PHPT, subclinical atherosclerosis could be halted by surgical treatment, whereas in patients with milder disease, who were not operated, subclinical atherosclerosis worsens over time.

Several previous studies on this matter investigated small samples including mixed populations in terms of age, gender, and menopausal status. Our results were obtained from a sample of consecutive patients with PHPT, larger than that of several previous studies (12-20, 24, 25). Moreover, the mean age was higher than that of other reports (12-16, 20, 23-25). In fact, in light of the known differences in the pathogenesis, epidemiology, and clinical features of CV diseases among men, premenopausal, and postmenopausal women (26), and considering the epidemiologic pattern of PHPT (27), we chose to investigate only female postmenopausal patients. This criterion obviously excludes the possibility of generalizing the current results to men and premenopausal women with PHPT, but the resulting picture more reliably reflects the real-world clinic. With the same purpose, although at variance with some previous papers (12-15, 18, 20, 23-25), we did not exclude smokers, or patients with diabetes mellitus, as well as those on antihypertensive or statin therapy.

According to some previous cross-sectional studies, the IMT of PHPT patients did not differ from that of control subjects (12, 13, 17, 19, 20), although 2 studies (12, 13) detected impaired flow-mediated vasodilation, indicating endothelial dysfunction, despite still unaffected structure. Other studies demonstrated higher IMT values in all PHPT patients (15, 17, 18, 23, 24) or only in PHPT patients with CV risk factors as diabetes mellitus, arterial hypertension, dyslipidemia, obesity, or smoking habits (16). Whichever the initial vascular involvement, we chose to prospectively investigate the possible progression of IMT and plaques in postmenopausal women with PHPT (ie, the most common presentation of the disease), managed according to the criteria dictated by the current consensus (28). In order to highlight such a vascular involvement, our patients were assessed after at least 24 months, at variance with most previous studies (13, 14, 17, 20, 23-25) that investigated patients for a shorter time lag, which could be inadequate to put into evidence significant variations.

Our most relevant finding is that of a diverging evolution of early-stage atherosclerotic pattern of patients who underwent surgical or conservative management. Noticeably, our 2 groups of PHPT patients did not differ for age, or all the other recognized CV risk factors, or IMT and plaque prevalence at baseline evaluation. Those who met criteria for surgery, indicating a more severe parathyroid hyperfunction, obviously had a postsurgical normalization of all parameters of PHPT, whereas they did not have significant change of IMT or varying plaque incidence after surgery. Instead, conservatively managed patients showed significantly higher IMT values at follow-up, not associated with increased incidence of plaque. Such results did not change after correcting for blood pressure values, which were significantly increased at the follow-up check in both PTx and non-PTx groups. These results, in line with most previous ones (13, 14, 17, 19, 20), suggest that parathyroidectomy could halt the progression of early atherosclerotic CV involvement in PHPT patients, despite the more severe parathyroid disease. Instead, our data suggest that an even milder parathyroid hyperfunction, if left untreated, could induce a worse evolution of the vascular disease. The unchanged frequency of atherosclerotic plaques at the follow-up of both groups probably relates to the time lag needed for plaque development, whereas IMT seems to allow an earlier identification of patients at risk for more advanced vascular damage (32). The longer follow-up time of PTx patients does not weaken, and actually reinforces the meaning of our current findings. Our study suffers from some limits, such as the relatively small sample size, the lack of generalizability to other groups of PHPT patients, and the lack of data on CV outcome. It is worth noting that due to the initial design, we did not analyze plaque size, quality, or architecture, which are all relevant prognostic factors affecting future CV risk. Strengths of the study include the inclusion criteria, which avoid possible bias related to age, gender, or menopausal status, the investigation of the main CV risk factors, the adequate follow-up duration, and the adoption of grouping criteria adherent to the real-life clinic.

In conclusion, our results suggest that early atherosclerotic involvement of the carotid wall progresses in untreated PHPT patients and not in those undergone parathyroidectomy. If confirmed on larger series and/or supported by CV outcome data, our results could even imply the need to rethink some principles of management of PHPT.

## Data Availability

Some or all datasets generated during and/or analyzed during the current study are not publicly available but are available from the corresponding author on reasonable request.

## Abbreviations

25(OH)D: 25-hydroxyvitamin D
CV: cardiovascular
eGFR: estimated glomerular filtration rate
HbA1c: glycosylated hemoglobin
HDL: high-density lipoprotein
HOMA-IR: homeostasis model assessment of insulin resistance
IMT: intima-media thickness
LDL: low-density lipoprotein
PHPT: primary hyperparathyroidism
PTH: parathyroid hormone
PTx: parathyroidectomy
